# Burden of Long COVID-19 in a Cohort of Recovered COVID-19 Patients in Delhi, India

**DOI:** 10.7759/cureus.60652

**Published:** 2024-05-20

**Authors:** Mongjam M Singh, Hitakshi Sharma, Nidhi Bhatnagar, Amod Laxmikant Borle, Shivani Rao, Suruchi Mishra, Gurmeet Singh, Tanya Singh, Mahima Kapoor, Naresh Kumar

**Affiliations:** 1 Community Medicine, Maulana Azad Medical College, New Delhi, IND; 2 Psychiatry, Maulana Azad Medical College, New Delhi, IND; 3 Pulmonary Medicine, Maulana Azad Medical College, New Delhi, IND

**Keywords:** persistent symptoms, sequelae, complications, covid-19, post covid syndrome, long covid syndrome

## Abstract

Background: The long COVID phase is characterized by signs and symptoms persisting for at least three months after recovery from acute COVID-19 illness. There is limited data on comprehensive long-term clinical follow-up of COVID-19 patients.

Aims: This study aims to explore the burden and symptomatology of long COVID syndrome and its association with various health parameters.

Settings and design: This prospective observational study was conducted in Delhi from May 2022 to March 2023.

Methods and material: A total of 553 adult patients who had recovered from COVID-19 were enrolled in the study. A sociodemographic and clinical profile was obtained using validated questionnaires, along with an evaluation of biochemical parameters to assess the associated factors.

Statistical analysis used: Chi-square test, unpaired t-test, and bivariate regression analysis were applied using Statistical Product and Service Solutions (SPSS, version 28; IBM SPSS Statistics for Windows, Armonk, NY). A p value of <0.05 was considered significant.

Results: A total of 252 patients (45.6%) had long COVID syndrome, which was significantly associated with the presence of any pre-existing comorbidity (OR=1.46 (1.02-2.09); p=0.039), previous history of hypertension (OR=1.82 (1.07-3.09); p=0.027), and vaccination against COVID-19 (OR=1.392 (1.171-1.656); p=0.003). The most common symptoms reported were persistent fatigue (33.3%) and persistent dry cough (28.5%). Patients with long COVID syndrome are also reported to have poorer sleep quality. Biochemical findings showed abnormal T lymphocytes (9.3%) and raised HbA1c (11.9%).

Conclusions: Multiple risk factors and symptoms associated with long COVID syndrome were identified in this study. Research efforts and knowledge regarding the pattern of illness will aid in long-term monitoring and development of interventional strategies and guidelines for the care of recovered COVID-19 patients.

## Introduction

Long COVID syndrome is defined as the collection of signs and symptoms in patients post-infection by the SARS-CoV-2 virus [1]. According to the World Health Organization (WHO), it is characterized by ongoing symptoms that persist for at least three months after the initial SARS-CoV-2 infection and cannot be explained by any other cause [2]. However, the Office for National Statistics in the UK estimated self-reported long Covid symptoms in 3%-11.7% after 12 weeks, impacting their social, professional, and day-to-day lives [3]. Additionally, long COVID may develop in nearly 20% of individuals infected with SARS-CoV-2 and encounter > 200 symptoms that can impact their daily activities [2].

The long COVID phase is characterized by multiple system involvement, ranging from asymptomatic or mild cases to severe respiratory distress and multi-organ failure [4]. The predictors of the rise and persistence of symptoms of COVID-19 infection are unclear, and pathophysiology is still ambiguous [5]. However, several different theories have been proposed to explain the etiology. One possibility is that the virus enters through the angiotensin receptor, which releases pro-inflammatory markers and cytokines that can lead to a hypercoagulable state and cytokine storm syndrome to affect multiple organs [5]. Still, the obscurity poses a major challenge for both patients and healthcare providers. Moreover, the overlapping symptoms, new emerging COVID strains, and lack of definitive treatment protocol have increased the perplexity associated with long COVID.

There has been limited exploratory research on long COVID syndrome with scarce data on long-term outcomes [6]. Hence, to develop appropriate management strategies, optimize healthcare delivery, and provide support to recovered COVID-19 patients in the community, it is important to understand the long-term effects of the virus. Previous studies have focused on specific populations or organ systems, reiterating the need to comprehensively assess the long-term consequence of the disease in a cohort of recovered patients [7]. We therefore planned to conduct this study to assess symptomatology, associated factors, and the impact on various health parameters of long COVID syndrome among recovered patients in Delhi.

## Materials and methods

Study design: This study is a cross-sectional part of a prospective observational study, which was conducted in Delhi among recovered COVID-19 patients, highlighting the important findings of the data. The study was approved by the institutional ethics committee (F.1/IEC/MAMC/87/05/2021/No526). The community-based study was carried out in 11 districts of Delhi. The data were collected from May 2022 to March 2023.

Participants: A list of COVID-19-diagnosed patients was received daily from the public health wing of Directorate Health Services, Government of National Capital Territory, Delhi, containing details of 10-15 COVID-19-positive patients, among which five to six patients were selected randomly by computer-generated random numbers and were contacted. If they fulfilled the study enrolment criteria and provided consent, they were included in the study. Recovered adult patients (>18 years) infected with COVID-19 for the first time were included in the study after 14 days of their diagnosis [8]. Pregnant women, patients with a history of re-infection of COVID-19, and patients with any terminal illness were excluded. A total of 553 patients were enrolled at the baseline of the study.

Data collection: After enrolment, the study team visited the participants at their residence, and their detailed history was obtained through medical records and interview schedules, including age, sex, education, socio-economic status, baseline comorbidity details, and treatment profile. Standardized and validated questionnaires were used to assess associated factors - WHO Case Report Form for Post-COVID Conditions, Depression, Anxiety and Stress Scale (DASS-21), WHO Quality of Life-BREF (WHOQOL BREF), Pittsburg Sleep Quality Assessment, and St. George’s Respiratory Questionnaire. Evaluation of biochemical parameters such as HbA1c levels, absolute neutrophil count, absolute eosinophil counts, and peripheral smear examination was also done. During data collection, 523 out of 553 participants consented to the evaluation for DASS-21 and WHOQOL-BREF questionnaires. Meanwhile, biochemical analysis was done for 525 participants because of the sample loss. Consultation by a general physician (physical/tele-consultation) was provided, and wherever required, participants were referred to a specialist for further investigations and tele-consultations.

Data analysis: The collected were was entered, cleaned, and coded in MS Excel and was analyzed in Statistical Product and Service Solutions (SPSS, version 28; IBM SPSS Statistics for Windows, Armonk, NY). The categorical variables were expressed as proportions, and continuous data were expressed as mean (SD) and median (IQR). A chi-square test was applied for statistical differences between categorical variables, and significant differences between means were tested using an unpaired t-test. Bivariate analysis was also done to assess the strength of association among categorical variables that showed significant association in preliminary analysis.

## Results

A total of 553 patients were screened out, with a mean age of 41.83 ± 14.9 years, among which 53% were males, 94.6% participants had never smoked, and 91% had no history of alcohol intake in the last three years. A similar distribution of characteristics was seen among 252 (45.6%) patients with long COVID syndrome. A total of 175 participants (31.6%) had at least one comorbidity, with hypertension (11.4%, 63) and obesity (11.6%, 64) being the most common. Moreover, 514 participants were vaccinated (92.9%), among which 472 participants (91.8%) had received two doses of the vaccine, as shown in Table 1.

**Table 1:** Demography and other factors associated with the symptoms of long COVID syndrome LCoVS - long COVID syndrome; df - degree of freedom; X^2^ - chi-square value

Category	Variables	LCoVS Absent, N=301, Number (%)	LCoVS Present, N=252, Number (%)	X^2^ (df)	P value
Sex	Male	169 (57.7)	124 (42.3)	2.65, (1)	0.10
	Female	132 (50.8)	128 (49.2)		
Age	18-30 years	87 (55.1)	71 (44.9)	1.47 (3)	0.689
	31-45 years	99 (55)	81 (45)		
	46-60 years	78 (51)	75 (49)		
	>61 years	37 (59.7)	25 (40.3)		
Smokers	Never smoker	284 (54.3)	239 (45.7)	6.78 (2)	0.034
	Current smoker	8 (40)	12 (60)		
	Past smoker	9 (90)	1 (10)		
Alcohol intake	Yes	22 (44)	28 (56)	3.21 (1)	0.20
Morbidity profile	Any comorbidity	84 (48)	91 (52)	4.27 (1)	0.039
	Hypertension	26 (41.3)	37 (58.7)	4.96 (1)	0.026
	Diabetes	21 (45.7)	25 (54.3)	1.559 (1)	0.212
	Obesity	31 (48.5)	33 (51.5)	1.05 (1)	0.31
	Cardiovascular disease	8 (57.2)	6 (42.8)	0.043 (1)	0.836
	Chronic lung disease	3 (33.3)	6 (66.7)	1.64 (1)	0.20
	Cancer	3 (75)	1 (25)	0.687 (1)	0.407
Vaccination status	Vaccinated	270 (52.5)	244 (47.5)	10.73 (2)	0.005
	Unvaccinated	18 (81.8)	4 (18.2)		
	Status unknown	13 (76.5)	4 (23.5)		
Doses of vaccination	1 Dose	21 (67.8)	10 (32.2)	13.49 (2)	0.001
	2 Doses	242 (51.3)	230 (48.7)		
	Not known	7 (63.6)	4 (36.4)		

In univariate regression analysis for the determinants of long COVID syndrome, having the presence of any pre-existing comorbidity (OR=1.46 (1.02-2.09); p=0.039) and previous history of hypertension (OR=1.82 (1.07-3.09); p=0.027) was found to be significantly associated risk factors of long COVID syndrome. Unvaccinated individuals were found to have higher odds of not having long COVID syndrome (OR=1.392 (1.171-1.656); p=0.005). However, in multivariate regression analysis, previous history of hypertension was found to be significantly associated with the presence of long COVID syndrome (aOR=1.40 (1.04-1.91)).

**Figure 1:**
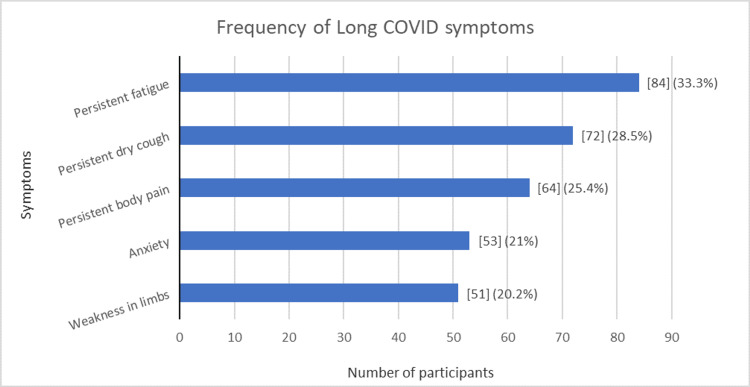
Frequency of five most commonly reported long COVID symptoms

Figure 1 shows that, among 252 participants reporting long COVID syndrome, persistent fatigue was the most common symptom reported by 84 (33.3%) participants, followed by persistent dry cough (28.5%). Trouble in concentration or forgetfulness (14.6%), chest pain (6.7%), and dizziness (5.1%) were key symptoms reported. Other symptoms, such as joint pain, persistent headache, shortness of breath, fever, post-exertion malaise, and weight loss were also reported. A single persistent symptom was reported among 107 participants (42.4%), whereas 56 reported more than one persistent symptom (22.2%).

St. George's Respiratory Questionnaire was used using scores (0-100) to assess the respiratory health of the participants. A higher score signifies bad respiratory health. While there was no significant difference (p=0.16) between the groups, more participants without long COVID syndrome had the best respiratory health (98%). Meanwhile, poorer respiratory outcomes were seen to be more prevalent among participants with long COVID syndrome.

Mental health

Table 2 describes that, among the study participants who reported the presence of long COVID syndrome, 6.8% reported mild depression, 12.2% reported moderate depression, 6.3% reported severe depression, and 9.3% reported extremely severe depression. Similarly, 5.5% reported mild anxiety, 17.3% moderate anxiety, 6.3% severe, and 16% extremely severe anxiety. Similar results were seen for stress scores. No significant difference was found among participants with or without long COVID syndrome.

**Table 2:** Association of depression, anxiety, and stress with long COVID syndrome (N=523) LCoVS - long COVID syndrome; df - degree of freedom; X^2^ - chi-square value; DASS 21 scale - Depression, Anxiety and Stress Scale

Scale Used		LCoVS Absent, N= 286 Number (%)	LCoVS Present, N= 237 Number (%)	X^2^ (df)	P value
DASS 21 Scale					
Depression (N= 523)	None	163 (57)	155 (65.4)	6.475 (4)	0.16
	Mild	16 (5.6)	16 (6.8)		
	Moderate	56 (19.6)	29 (12.2)		
	Severe	21 (7.3)	15 (6.3)		
	Extremely severe	30 (10.5)	22 (9.3)		
Anxiety (N=523)	None	153 (53.4)	130 (54.9)	8.723 (4)	0.068
	Mild	12 (4.2)	13 (5.5)		
	Moderate	31 (10.8)	41 (17.3)		
	Severe	30 (10.4)	15 (6.3)		
	Extremely severe	60 (21)	38 (16)		
Stress (N=523)	None	192 (67.1)	170 (71.7)	2.742 (4)	0.602
	Mild	34 (11.9)	26 (11)		
	Moderate	15 (5.2)	14 (5.9)		
	Severe	40 (14)	25 (10.5)		
	Extremely severe	5 (1.8)	2 (0.8)		

The Pittsburgh Sleep Quality Index was used for sleep assessment, and 78.2% of participants reported that long COVID syndrome had good sleep compared to 89% of participants without long COVID. The difference between the groups was statistically significant (p<0.001).

Quality of life

WHOQOL-BREF (Australian version) was used to assess the quality of life under four domains, as shown in Table 3. It was found that median scores of physical health (p=0.47), psychological health (p=0.75), social relationship (p=0.65), and environmental health (p=0.78) of participants with or without long COVID syndrome were not statistically significant.

**Table 3:** Association of quality of life scores with long COVID syndrome (N=523) LCoVS - Long COVID syndrome; WHOQOL-BREF - World Health Organization Quality of Life Brief Version; * using Mann-Whitney U test

Median Scores of the WHOQOL-BREF Questionnaire	LCoVS Absent, N=286, Number (%)	LCoVS Present, N=237, Number (%)	P value*
Physical Health (Domain 1)	68 (61-79)	68 (61-79)	0.473
Psychological Health (Domain 2)	75 (63-88)	75 (63-88)	0.749
Social Relationships (Domain 3)	75 (67-92)	75 (67-92)	0.649
Environmental Health (Domain 4)	75 (69-71)	78 (66-91)	0.778

Biochemical parameters

Glycosylated hemoglobin (HbA1C) analysis was done in 513 participants, among which 71.7% participants had normal HbA1C, while 11.9% had raised HbA1c > 6.5 (rest had HbA1c levels below the normal range). When compared among study participants with and without long COVID, no significant difference was found (p=0.20). Abnormalities were found in peripheral smear examination where 43 out of 525 participants (8.2%) had transformed lymphocytes and 16 (3%) had activated monocytes. Out of 525 participants, 100 (19%) had elevated absolute neutrophil count, and 42 (8%) had elevated absolute eosinophil count.

## Discussion

Our study provides valuable insights into the clinical presentation and factors associated with long COVID syndrome. It was found that 45.6% of participants reported the persistence of symptoms even after four weeks of being diagnosed with COVID-19 infection. There was no significant difference between the age groups of participants with and without long COVID syndrome. However, studies conducted by Emecen et al. [9] and Kostev et al. [10] reported that age >40 years is significantly associated with the presence of long COVID.

Our study reported smoking to be significantly associated with long COVID (p=0.034), which is consistent with the studies conducted by Jones et al. [11], Silverberg et al. [12], and Peterson et al. [13]. Established damage to the respiratory system in participants who were already smoking may be the reason for the development of long COVID symptoms. In our study, pre-existing co-morbidities (OR=1.46; 95% CI=1.02-2.09) and a previous history of hypertension (OR=1.82; 95% CI=1.07-3.09) were found to be a significant risk factor of having long COVID. Similar findings were seen in the study conducted in India by Arjun et al. [14] where the adjusted odds ratio (aOR) of having pre-existing comorbidity was higher among participants with long COVID syndrome (aOR=2.00; 95% CI=1.16-3.44). Pre-existing hypertension was also a risk factor in another study conducted by Tleyjeh et al. [15] in 2020 (aHR=1.73; 95% CI=1.09-2.74).

In the present study, vaccination against COVID-19 was associated with the presence of long COVID, as also reported by Tsuchida et al. [16], which contradicts the studies conducted by Byambasuren et al. [17] and Davis et al. [18]. Previous vaccination could be protective for the development of symptoms after recovery from acute COVID-19 illness. However, the development of these symptoms from acute illness and vaccination could not be distinguished.

Additionally, 42.4% of participants in our study reported the presence of at least one symptom where commonly reported symptoms were persistent fatigue (33.3%), persistent dry cough (28.5%), and persistent body pain (25.4%) among others. A similar study conducted in the UK in 2022 found that, at three, six, and nine months, 62%, 52%, and 49% of patients had reported long COVID symptoms, respectively, among which breathlessness, fatigue, and cough were the most common [19]. Another study conducted in Norway found that 55% of the total home-isolated participants had persistent symptoms six months after initial COVID infection and the commonest symptom was fatigue, followed by difficulty in concentration and reduced smell/taste [20]. Another study conducted in India by Arjun et al. found the prevalence of long COVID to be 29.2%, with fatigue and cough being the commonly reported symptoms [14]. Deterioration of the immune system after acute viral illness might be the reason for persistent fatigue, whereas the targeted impact on the respiratory system by the COVID-19 virus might be the reason for cough and other respiratory symptoms.

In the present study, the proportion of participants reporting depression, anxiety, and stress was higher among participants with long COVID syndrome, which might be due to the involvement of the hypothalamus during acute illness. Similar results were reported by Asio et al. [21] and Menges et al. [22]. Though the differences between the groups were not significant, it certainly stresses the need for further diagnosis and interventions for the long-term mental health of patients who recovered from COVID. The quality of sleep was poorer among participants with long COVID, which might be due to the involvement of the hypothalamus during acute illness. This was consistent with the findings reported by Chhajer et al. [23] from India and Ayesha et al. [24] from Pakistan.

There was no significant association found between quality of life scores and the presence of long COVID syndrome using the WHOQOL-BREF questionnaire. However, studies conducted by Sarda et al. [25] reported that patients with long COVID had a significantly lower quality of life compared to those without long COVID (mean difference=-0.24; 95% CI=-0.31 to -0.17; p<0.001). Similar findings were reported by Hawlader et al. from Bangladesh (mean score=64.15) [26]. Our findings might be due to the adaptation of mitigation strategies and coping mechanisms among study participants, as well as their improved sense of well-being.

Limitations

The key strength of the study was that it was the first study of its kind conducted in the study area. The study integrated extensive consultation from clinicians and a variety of symptoms were analyzed. The study was limited in generalizability due to the small sample size in consideration. The period of data collection coincided with the onset of the OMICRON wave and its lineage in the country. The long COVID effects observed might vary depending on the strain in circulation. The pre-COVID status of patients was not available to the investigators, and thus correlations with their baseline status cannot be drawn. Moreover, the study might be influenced by high vaccination coverage in the population and many subtle post-vaccination effects in the study participants. There might be cases of hidden COVID reinfection in the study population as the trend of getting tested for COVID-19 was declining in the latter half of the pandemic. The same might interfere with the observed study findings.

## Conclusions

Patients with acute COVID-19 illness require long-term follow-up after recovery. Multiple factors associated with the presence of persistent symptoms of long COVID have been identified in this study. Patients who are smokers and with co-morbidities require special attention post recovery from acute COVID-19 illness. Longer follow-up studies in a larger population are warranted to comprehend the persistence and incidence of symptoms due to long COVID syndrome, which will aid in the development of guidelines for clinical management and care of individuals with long COVID.
